# A Label-Free Droplet Sorting Platform Integrating Dielectrophoretic Separation for Estimating Bacterial Antimicrobial Resistance

**DOI:** 10.3390/bios14050218

**Published:** 2024-04-26

**Authors:** Jia-De Yan, Chiou-Ying Yang, Arum Han, Ching-Chou Wu

**Affiliations:** 1 Doctoral Program in Tissue Engineering and Regenerative Medicine, National Chung Hsing University, Taichung City 402, Taiwan; e168gg@gmail.com; 2 Institute of Molecular Biology, National Chung Hsing University, Taichung City 402, Taiwan; cyyang@dragon.nchu.edu.tw; 3 Department of Electrical and Computer Engineering, Texas A&M University, College Station, TX 77843, USA; 4 Department of Biomedical Engineering, Texas A&M University, College Station, TX 77843, USA; 5 Department of Chemical Engineering, Texas A&M University, College Station, TX 77843, USA; 6 Department of Bio-Industrial Mechatronics Engineering, National Chung Hsing University, Taichung City 402, Taiwan; 7 Innovation and Development Center of Sustainable Agriculture, National Chung Hsing University, Taichung City 402, Taiwan

**Keywords:** droplet microfluidics, dielectrophoretic (DEP) sorting, antimicrobial resistance

## Abstract

Antimicrobial resistance (AMR) has become a crucial global health issue. Antibiotic-resistant bacteria can survive after antibiotic treatments, lowering drug efficacy and increasing lethal risks. A microfluidic water-in-oil emulsion droplet system can entrap microorganisms and antibiotics within the tiny bioreactor, separate from the surroundings, enabling independent assays that can be performed in a high-throughput manner. This study presents the development of a label-free dielectrophoresis (DEP)-based microfluidic platform to sort droplets that co-encapsulate *Escherichia coli* (*E. coli*) and ampicillin (Amp) and droplets that co-encapsulate Amp-resistant (AmpR) *E. coli* with Amp only based on the conductivity-dependent DEP force (*F_DEP_*) without the assistance of optical analyses. The 9.4% low conductivity (LC) Luria–Bertani (LB) broth diluted with 170 mM mannitol can maintain *E. coli* and AmpR *E. coli* growth for 3 h and allow Amp to kill almost all *E. coli*, which can significantly increase the LCLB conductivity by about 100 μS/cm. Therefore, the AmpR *E. coli*/9.4%LCLB/Amp where no cells are killed and the *E. coli*/9.4%LCLB/Amp-containing droplets where most of the cells are killed can be sorted based on this conductivity difference at an applied electric field of 2 MHz and 100 V_pp_ that generates positive *F_DEP_*. Moreover, the sorting ratio significantly decreased to about 50% when the population of AmpR *E. coli* was equal to or higher than 50% in droplets. The conductivity-dependent DEP-based sorting platform exhibits promising potential to probe the ratio of AmpR *E. coli* in an unknown bacterial sample by using the sorting ratio as an index.

## 1. Introduction

Antimicrobial resistance (AMR) in bacteria, viruses, fungi, and parasites has become a global health issue and seriously threatens public health. Antibiotic abuse is one of the reasons that causes growth in AMR, which increases the costs of clinical treatments and prolongs recovery periods [1]. Antimicrobial susceptibility testing (AST) and nucleic acid amplification testing (NAAT) are the two most commonly used methods to estimate bacterial AMR. Conventional microbiology cultures, such as disk-diffusion susceptibility testing and the antimicrobial gradient diffusion method, are the most popular AST methods [2]. However, the culture-based AST methods are time-consuming and labor-intensive. NAATs can specifically identify the AMR gene fragments of bacteria [3], but the required procedures and instruments for nucleic acid extraction and amplification limit the use of NAATs in high-throughput detection, especially when the sample volume is small. Developing a high-throughput microfluidic platform for rapid AMR bacterial detection can significantly impact public health.

Microfluidic systems can be used to generate sub-nanoliter droplets as discretized bioreactors to perform chemical and biological reactions within the droplets [4]. Droplet-based microfluidic systems have the advantages of low reagent consumption, high-throughput assay, and easy integration with automatic systems [5,6]. Through various droplet-manipulated techniques, including generation, merging, and sorting [7], droplet microfluidic chips have become powerful tools for enzymatic kinetic analysis [8,9,10,11], cellular metabolite monitoring [12,13,14,15], and antibiotic assay [16,17,18]. Among the droplet-based manipulation steps, sorting target droplets from a mixture is critical for subsequent analysis. For example, sorting droplets based on cell or microorganism growth status is meaningful for antibiotic susceptibility, anticancer drug screening, and biomedical diagnostics [19,20].

The sorting criteria of droplets usually depend on the droplet’s size, conductivity, or optical properties. Electric sorting techniques, especially dielectrophoresis (DEP), exhibit great feasibility in being integrated into microfluidic chips for high-throughput manipulation of target samples [21]. DEP force (*F_DEP_*) is derived from the net dipole moments of a particle induced by a non-uniform electric field. The positive *F_DEP_* (*F_pDEP_*) can drive the more polarized droplets than the surrounding medium to a high electric field region and vice versa for the negative *F_DEP_* (*F_nDEP_*) [22]. The DEP behavior of droplets depends on the difference in conductivity and dielectric permittivity between the droplets and the medium at the low and high frequencies, respectively [23]. When the droplet’s conductivity exceeds the medium at frequencies lower than the crossover frequency, *F_pDEP_* can drive the droplets to the high electric field region.

Most DEP-assisted microfluidic sorting systems require optical detectors, such as fluorescent detectors [24,25,26], ultraviolet-visible (UV-vis) spectroscopes [27,28], Raman spectroscopes [29], and light-scattering microscopes [30] equipped with image processing software [31] to distinguish target droplets, based on which the DEP field can be switched on or off for the sorting procedures. Fluorescent probes and antibody-coated beads are commonly used to recognize target droplets. For example, Cole et al. utilized a fluorescence-activated DEP-based microfluidic sorter to selectively dispense single-cell droplets on an oil-covered and motorized substrate [32]. Furthermore, Liu et al. designed a microfluidic chip with a laser light-scattering detector for sorting AMR bacteria. After culturing mutated *Escherichia coli* (*E. coli*) in droplets for 13 h, the mutated *E. coli* still proliferated to cause significant light scattering as an optical signal to switch DEP on for sorting the droplets in the downstream microchannel [30]. Although optical judgment and DEP manipulation can facilitate precise droplet sorting, the requirement of expensive optical instruments, a complicated control system, or fluorescent labels adds to the burden of utilizing this method in many practical settings.

In contrast, size- and conductivity-dependent DEP actuation have promising potential to establish a label-free sorter without the need for optical detectors. Notably, the conductivity-dependent *F_DEP_* can separate two conductivity-varied particles with the same size at a specific frequency by adjusting the surrounding medium conductivity to induce *F_pDEP_* or *F_nDEP_* [33,34]. Unfortunately, changing the oil conductivity in the water-in-oil droplet microfluidic platform is challenging due to the low ionic solubility. Therefore, the frequency-dependent DEP method becomes a holy grail for sorting the conductivity-varied water-in-oil droplets resulting from the droplet’s *F_DEP_* spectrum. To the best of our knowledge, the frequency-based *F_pDEP_* method has not been applied for sorting conductivity-varied water-in-oil emulsion droplets in a microfluidic chip for detecting AMR bacteria.

This study proposes a label-free *F_pDEP_*-based droplet-sorting platform for estimating the existence of AMR bacteria in samples. *E. coli* and AMR *E. coli* were used in conjunction with ampicillin (Amp) antibiotics to demonstrate the platform’s feasibility. When the bacteria encapsulated in water-in-oil emulsion droplets are susceptible to antibiotics, the destroyed bacterial cells leak a highly conductive cytoplasm to the droplet medium, increasing the droplet conductivity, which results in a larger *F_pDEP_* than the droplets containing antibiotic-resistance bacteria. Using these differences, the *F_pDEP_*-based microfluidic platform could sort droplets containing antibiotic-susceptible *E. coli* from droplets containing AMR *E. coli*. Furthermore, the frequency and magnitude of the adequate *F_pDEP_* for sorting conductivity-varied droplets are discussed in detail.

## 2. Materials and Methods

### 2.1. Theory of DEP

The DEP phenomenon describes the polarization of neutral-electricity particles suspended in a medium induced by non-uniform electric fields. The *F_DEP_* of a spherical particle can be presented as Equation (1) [35]:

(1) $$F_{DEP}=2\pi \varepsilon_{m}r^{3}Re(f_{CM})\nabla E^{2}$$

where *ε_m_* represents the relative permittivity of the surrounding medium, *r* is the particle’s radius, Re(*f_CM_*) is the real part of the Clausius–Mossotti (CM) factor, and *E* stands for the amplitude of the electric field. When two droplets have the same *r* in the same medium and electric field, Re(*f_CM_*) determines the *F_DEP_* vector of particles. The *f_CM_* represents the particle’s polarizability, as described in Equation (2) [33]:

(2) $$f_{CM}=\frac{\varepsilon_{p}^{*}-\varepsilon_{m}^{*}}{\varepsilon_{p}^{*}+2\varepsilon_{m}^{*}}$$

where ε*_p_ and ε*_m_ represent the complex permittivity of the particle and the medium, respectively, and ε* = ε − *i*(σ/ω), where σ is the conductivity and ω(=2π*f*) is the angular frequency. When the ω varies across the relaxation frequency (*f_rex_*), as mentioned in Equation (3) [23], the *f_CM_* changes sign, indicating a transition from pDEP to nDEP, or vice versa.

(3) $$f_{rex}=\frac{1}{2\pi }\frac{\sigma_{p}+2\sigma_{m}}{\varepsilon_{p}+2\varepsilon_{m}}$$

In other words, the *F_pDEP_* declines with increasing frequencies before reaching the *f_rex_*. Thus, in the case of droplets, as the conductivity of the droplets increases, the *F_pDEP_* becomes larger and *f_rex_* becomes higher in the same medium. Therefore, a fixed frequency can produce different *F_pDEP_* between two droplets having different conductivity in the same surrounding oil.

### 2.2. Design and Fabrication of the Droplet Sorter

**Figure 1:**
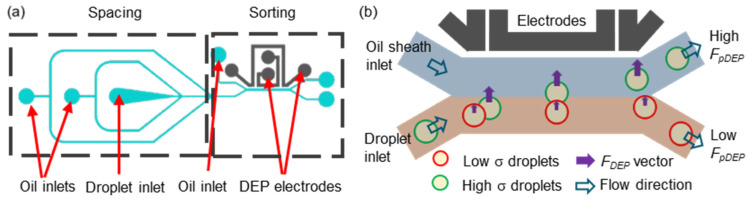
Schemes of the droplet sorter chip (**a**) and the *F_pDEP_* effect on the conductivity (σ)-varied droplets in the non-homogeneous electric field (**b**). High σ droplets experience a greater *F_pDEP_* than low σ droplets with a longer movement perpendicular to the flow streamline and are collected in the high *F_pDEP_* channel.

The microchannels of the droplet generator and sorter were made from polydimethylsiloxane (PDMS) (PM5040, Bingbond Co., Ltd., Tainan City, Taiwan) by the soft lithography procedure. The microchannel’s dimension of the generator and sorter chips is marked in Figure S1 (Supplementary Information). The patterned negative photoresist layers, consisting of a SU-8 3010 (MicroChem, Newton, MA, USA) photoresist layer (10 μm) spin-coated on a cleaned glass substrate and a dried SU-8 film (100 μm) (SUEX K100, DJ Mircolaminates, Inc., Sudbury, MA, USA) attached to the SU-8 3010 layer at 70 °C, were used as a mold for fabricating the PDMS replica. After performing exposure and development, PDMS was poured on the SU-8 mold and peeled from the SU-8 mold-containing glass substrate after curing at 75 °C for 1 h. Inlets and outlets were punched using a hollow cylinder-shaped hole puncher. Oxygen plasma was utilized to bond the microchannel-containing PDMS slab on a cleaned glass substrate to form the chips of the droplet generator and sorter. Figure 1a shows the design of the droplet sorter, including the droplet spacing and sorting components. To form the 3D DEP electrodes, the droplet sorter chips were heated on a hotplate at 75 °C, and the DEP electrode channels were filled with low-melting-point (70 °C) bismuth alloy (Ultimate Materials Technology Co., Ltd., Hsinchu County, Taiwan). Instantly, wires were inserted into the inlets of the DEP electrode channels as interconnects to a function generator. The geometric design of the 3D DEP electrode pair and the sorting channel similar to that shown in the work by Simon et al. [36] results in a significantly high effective DEP range twice as large as the DEP electrode height. In this study, the distance from the midline of the sorting channel to the DEP electrode is 190 μm. Since the electrode height is 110 μm, this implies that the effective DEP field can sufficiently cover a large portion of the droplet in the DEP-sorting channel.

The working mechanism of the *F_pDEP_*-based droplet sorter is determined by the droplet movement perpendicular to the fluidic flow direction in the sorting channel, which depends on the *F_pDEP_* magnitude. Figure 1b shows the sorting mechanism. The higher σ droplets, like the *E. coli*/Amp-containing droplets, experience a stronger *F_pDEP_* and thus move to the high *F_pDEP_* (H*F_pDEP_*) channel. The Amp-resistance (AmpR) *E. coli*/Amp-containing droplets having lower σ undergo the weaker F_pDEP_ to have a smaller perpendicular movement, thus being collected in the low *F_pDEP_* (L*F_pDEP_*) channel.

### 2.3. Bacterial Culture

*E. coli* (HB101) and AmpR *E. coli* with the pET21b plasmid (Amp resistance gene) [37] were cultured in Luria–Bertani (LB) broth (BD 244620, Becton, Dickinson and Company, Franklin Lakes, NJ, USA), consisting of 10 g/L tryptone, 5 g/L yeast extract, and 0.085 mol/L sodium chloride, at 37 °C with 150 rpm shaking overnight. The cell density was detected using a spectrophotometer (CT-2300, Chrom Tech, Inc., Apple Valley, MN, USA) at 600 nm to dilute the *E. coli* concentrations to 1 optical density (OD). Using 170 mM mannitol solution to replace 85 mM NaCl of LB broth as the low conductivity (LC) LB broth maintains the osmotic pressure and lowers the broth’s conductivity. After centrifugation and supernatant removal, the residual *E. coli* was washed using 170 mM mannitol and re-suspended at about 2 × 10^8^ CFU/mL density in the concentration-varied LCLB broths diluted with 170 mM mannitol and spiked with or without 100 μg/mL Amp (A9518, Sigma-Aldrich, St. Louis, MO, USA). Subsequently, the *E. coli* samples were cultured at 37 °C with 150 rpm shaking for 3 h to detect the solution conductivity change and bacterial growth status.

### 2.4. Measurement of Solution Conductivity and Bacterial Growth Rate

A four-electrode chip was fabricated to measure the conductivity of the microliter-scale bacterial culture medium before and after the 3-hour culture. After centrifugation, the supernatants of the samples (20 μL) were dripped onto the chip and an LCR meter (4284A, Agilent Technologies, Santa Clara, CA, USA) was utilized for conductivity measurements. The chip design and the conductivity measurements are described in S1 and Figure S2 (Supplementary Information).

The spread-plate culturing method was used to count the colony numbers to confirm the effect of the LCLB concentration and Amp antibiotics on bacterial growth. The 3-hour cultured *E. coli* suspension was diluted by a factor of 10^5^ with phosphate buffer saline for inoculation on LB agar (1.5%)-coated Petri dishes for 20 h for colony counting. All the mediums were autoclaved at 120 °C for 30 min before use.

### 2.5. Droplet Generation and Sorting

After subculture in concentration-varied LCLB for 3 h, the *E. coli* samples were loaded in a 1 mL syringe and injected into a flow-focusing microfluidic chip at a 50 μL/h flow rate. Fluorinated oil (Novec™-7500 (Sphere Fluidics, Cambridge, UK) with 1% Pico-Surf™ surfactant) was simultaneously pumped as a continuous phase flow into the microchannel at a 150 μL/h flow rate by syringe pumps (SPLab01, Baoding Shenchen Precision Pump Co., Ltd., Baoding, China) for droplet generation. Subsequently, the droplets were reflowed into the droplet inlet of the spacing component of the sorter chip (Figure 1a) at a 3 μL/h flow rate. The two oil inlets of the spacing component were injected with the fluorinated oil at a 30 μL/h flow rate to sufficiently space the sequential droplets. Simultaneously, the fluorinated oil was injected into the oil inlet of the sorting component as the sheath flow at a 70 μL/h flow rate for droplet sorting. A function generator (33220A, Agilent Technologies, Santa Clara, CA, USA) connected to a power amplifier (HA-405, Pintek Electronics Co., Ltd., New Taipei City, Taiwan) was employed to an output frequency-varied (1 to 3 MHz) 100 V_pp_ voltage for inducing DEP. The actual output voltage was monitored by an oscilloscope (DS1052E, Rigol Technologies, Suzhou New District, China).

## 3. Results and Discussion

### 3.1. Change in E. coli Growth Ratio and LCLB Conductivity after Culture

This study proposes a DEP-based microfluidic chip to sort conductivity-varied droplets containing *E. coli* and antibiotics. The AmpR *E. coli* can resist Amp, but standard *E. coli* are destroyed by Amp due to the Amp attachment to penicillin-binding proteins that interfere with cell wall peptidoglycan synthesis [38]. The LCLB concentration is critical to *E. coli* proliferation, allowing Amp inhibition on synthesizing the incomplete cell wall to cause cytoplasm leakage. In contrast, a low LCLB concentration may stop *E. coli* proliferation, which delays and reduces Amp action on the cell wall deterioration of *E. coli*.

**Figure 2:**
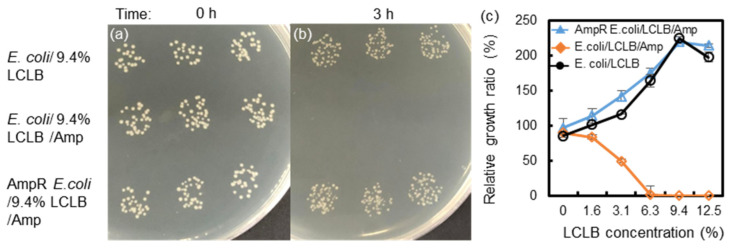
*E. coli* and AmpR *E. coli* in 9.4% LCLB broths without or with 100 μg/mL Amp (**a**) before and (**b**) after 3-hour culture and spotted on LB agar plates. (**c**) Relative growth ratios of bacteria counted from the colony number before and after the 3-hour culture in concentration-varied LCLB broths. Each value was calculated from three repetitions.

The spread-plate culturing method was used to count the colony numbers to confirm the effect of the LCLB concentration and Amp antibiotics on bacterial growth. Figure 2a,b show the bacterial colony images after spotting the 10^5^-diluted bacterial suspension on the agar-coated dishes for 20 h. The results show that the colony number of the *E. coli* cultured in 9.4% LCLB and the AmpR *E. coli* cultured in 9.4% LCLB/Amp increased, but the colony number of the *E. coli* cultured in 9.4% LCLB/Amp decreased to zero.

Figure 2c shows the bacteria’s relative growth ratios (defined as the number of colonies obtained from the 3-hour suspension culture divided by the number of colonies obtained from the 0-hour culture) versus the LCLB concentrations. The results show that the relative growth ratios of *E. coli* cultured in LCLB and AmpR *E. coli* cultured in LCLB/Amp increase with an LCLB concentration of 0−9.4%, implying that the higher the nutrient concentration of the broths is the more beneficial they become for *E. coli* and AmpR *E. coli* proliferation. These two samples’ relative growth ratios reach a plateau at the 9.4% LCLB concentration, indicating the maximal proliferation rates. Moreover, 0% LCLB (only 170 mM mannitol) is slightly adverse to bacterial growth, causing the growth ratio to decrease to 85.4% for *E. coli* and 96.5% for AmpR *E. coli*. In contrast, the relative growth ratios of *E. coli*/LCLB/Amp are inversely proportional to an LCLB concentration of 0−6.3%, indicating that Amp inhibits the cell wall synthesis of *E. coli* during proliferation to decrease *E. coli* viability. Interestingly, almost all *E. coli* were lysed in the LCLB/Amp higher than 6.3%, indicating that the bactericidal effect of ampicillin mainly acts on the growing *E. coli* with active cell wall synthesis [38].

**Figure 3:**
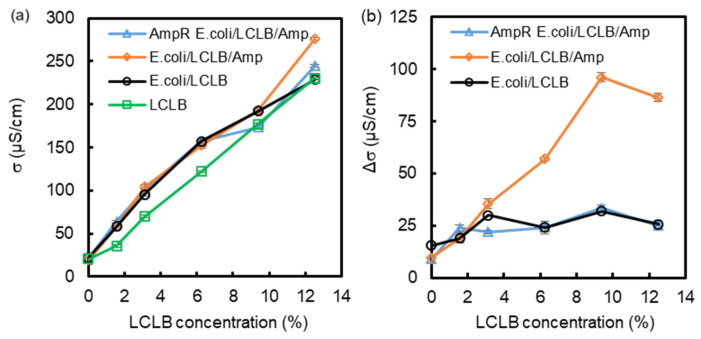
(**a**) Initial conductivities (σ) of the LCLB broths, the *E. coli*/LCLB mixture, the *E. coli*/LCLB/Amp mixture, and the AmpR *E. coli*/LCLB/Amp mixture before the 3-hour culture. (**b**) Conductivity increment (Δσ) of supernatants after the 3-hour culture. Each value was calculated from five repetitions.

The conductivities of the concentration-varied LCLB broths and the bacteria/LCLB/Amp mixtures were measured to analyze the contribution of *E. coli* growth and Amp inhibition to the solution before and after the 3-hour culture. Figure 3a shows the initial conductivities of the LCLB and the bacteria/LCLB/Amp mixtures before the 3-hour culture, where the LCLB conductivity linearly increased with increasing LCLB concentration. The conductivity of the bacteria/LCLB/Amp mixtures showed increasing trends similar to the LCLB conductivity while being around 22−35 μS/cm higher, which was attributed to the original LB broth residue. Furthermore, the overall conductivity fluctuation of the bacteria/LCLB/Amp mixtures was attributed to the operational variation between the different batches.

Figure 3b shows the conductivity changes after the 3-hour culture. The result shows that only the conductivity of the *E. coli*/LCLB/Amp mixtures increased with increasing LCLB concentration, reaching a maximum at 9.4% LCLB, and then slightly decreased at 12.5% LCLB concentration. The phenomenon is similar to the growth ratio seen in Figure 2c. The higher LCLB concentration causes more *E. coli* proliferation, allowing Amp inhibition to destroy the proliferated *E. coli*. The increments of conductivities are derived from the cytoplasm leakage due to the *E. coli* lysis [39].

In contrast, the conductivities of the AmpR *E. coli*/LCLB/Amp mixtures and the *E. coli*/LCLB mixtures had about a 25 μS/cm increment. These increases were independent of the LCLB concentration, indicating that the bacterial proliferation and metabolite in the concentration-varied LCLB cannot drastically increase the solution conductivity. Based on this result, the 9.4% LCLB condition was selected for the droplet study due to the maximum conductivity increment after the 3-hour culture under this condition, as this will result in the largest droplet-sorting efficiency in the *F_pDEP_*-based microfluidic chip.

### 3.2. Sorting Ratios of Conductivity-Varied Droplets

**Figure 4:**
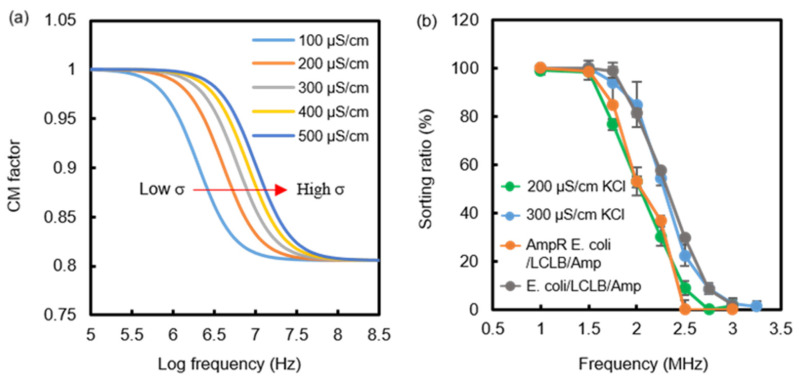
(**a**) The CM factor spectrum of conductivity-varied (100 to 500 μS/cm) droplets suspended in a fluorinated oil (4.5 nS/cm) calculated by the MyDEP software v1.0.1. (**b**) The sorting ratios (droplet number sorted into the H*F_pDEP_* channel divided by the total droplet number) of different droplets containing 200 μS/cm KCl, 300 μS/cm KCl, *E. coli*/9.4%LCLB/Amp, and AmpR *E. coli*/9.4%LCLB/Amp when applying 100 V_pp_ *F_pDEP_* in different frequencies. Each value was calculated from three repetitions in droplet-sorting experiments.

The *F_DEP_* magnitude of the same size droplets is dominated by the Re(*f_CM_*), as shown in Equation (1). The CM factor of droplets can be computed using the MyDEP (https://mydepsofteare.github.io, accessed on 11 January 2019) software published by Cottet et al. [33]. Figure 4a shows the CM factor spectrum of 100−500 μS/cm droplets suspended in fluorinated oil, which declines from 1 to 0.8 with increasing frequencies. The CM factor spectra show magnitude dispersion above zero due to the extremely low conductivity (4.5 nS/cm) and low relative permittivity (ε_r_ = 5.8) of the fluorinated oil. Moreover, the initial dispersion frequency increases with the droplet’s conductivity. Therefore, in choosing a fixed frequency in the dispersion window, one can obtain different CM factors for sorting conductivity-different droplets.

Four kinds of solutions, including 200 μS/cm KCl, 300 μS/cm KCl, the 3-hour cultured *E. coli*/LCLB/Amp mixture, and the 3-hour cultured AmpR *E. coli*/LCLB/Amp mixture were used for droplet generation and sorting. The droplet sorting was executed using frequency scans from 1 MHz to 3 MHz to obtain different *F_pDEP_* and sorting ratios, defined as the ratio of the droplet number collected in the H*F_pDEP_* channel to the total droplet number (*n* ≥ 60) passing through the DEP region in 1 min. Figure 4b shows the corresponding sorting ratio curves. The results show that the sorting ratios of the AmpR *E. coli*/LCLB/Amp-containing droplets were similar to those of the 200 μS/cm-KCl-containing droplets due to their similar conductivity.

Moreover, the same sorting trends occurred between the *E. coli*/LCLB/Amp-containing droplets and the 300 μS/cm-KCl-containing droplets. When the frequencies were lower than 1.5 MHz, the four types of droplets experienced a strong enough *F_pDEP_* to have a 100% collection efficiency into the H*F_pDEP_* channel. Although the sorting ratios of all the curves progressively decreased from 1.5 to 2.5 MHz due to the declining CM factors, the sorting ratio difference between the *E. coli*/LCLB/Amp-containing droplets and the AmpR *E. coli*/LCLB/Amp-containing droplets obtained at 2 MHz could reach 28.5%. This sorting ratio difference can be regarded as an index to estimate the AmpR *E. coli* ratio in an unknown bacterial suspension.

**Figure 5:**
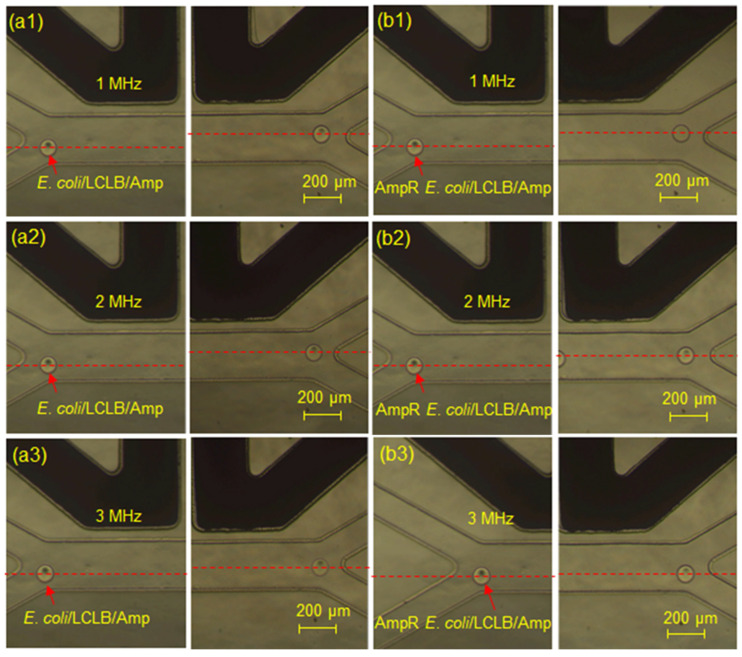
Position images of the (**a1**–**a3**) *E. coli*/LCLB/Amp and the (**b1**–**b3**) AmpR *E. coli*/LCLB/Amp-containing droplets passing through the *F_pDEP_* region of 1 (**a1**,**b1**), 2 (**a2**,**b2**), and 3 (**a3,b3**) MHz. The red arrows indicate the position of droplets. The red dashed lines indicate the central position of the droplets.

Figure S3 shows the trajectory images of an AmpR *E. coli*/LCLB/Amp-containing droplet in the DEP electrode region to demonstrate the *F_pDEP_* action of 1 MHz and 100 V_pp_ on the droplet with perpendicular displacement from the droplet inlet channel to the H*F_pDEP_* channel. Furthermore, Figure 5a1–a3,b1–b3 show *E. coli*/LCLB/Amp-containing droplets and AmpR *E. coli*/LCLB/Amp-containing droplets at the initial and final positions of the DEP electrode region with the 100 V_pp_ of 1, 2, and 3 MHz, respectively. Theoretically, to be sorted into the H*F_pDEP_* outlet, the droplets’ central position needs to vertically shift more than 45 μm from the droplet inlet channel across the midline of the sorting channel. The perpendicular displacements of *E. coli*/LCLB/Amp-containing droplets (*n* = 10) induced by 1, 2, and 3 MHz *F_pDEP_* were 73.1 ± 1.9, 50.8 ± 3.8, and 35.9 ± 2.9 μm, respectively. The phenomenon indicates that a lower frequency produces a stronger *F_pDEP_* to drag a droplet with a more extended perpendicular displacement. Similarly, the perpendicular displacements of AmpR *E. coli*/LCLB/Amp-containing droplets (*n* = 10) induced by 1, 2, and 3 MHz *F_pDEP_* were 70.5 ± 2.2, 45.2 ± 2.7, and 17.9 ± 2.2 μm, respectively, which are smaller than those of the *E. coli*/LCLB/Amp-containing droplets due to the smaller *F_pDEP_*. To summarize Figure 4b and Figure 5, the larger the perpendicular placement of the droplets is, the higher the sorting ratio.

### 3.3. Sorting Ratios of the AmpR E. coli-Mixed Samples

**Figure 6:**
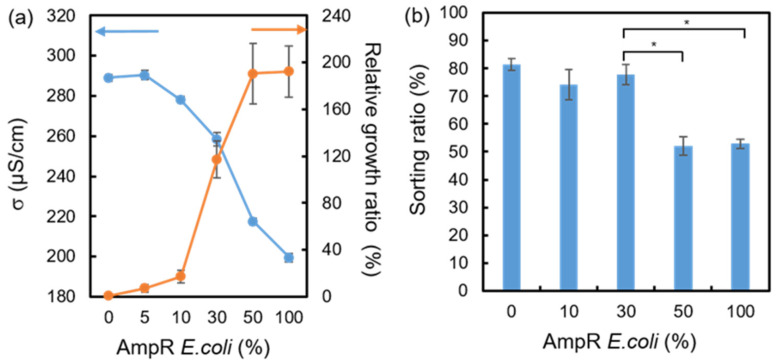
(**a**) Conductivities (σ, blue line) and relative growth ratios (orange line) obtained from the ratio-varied AmpR *E. coli* bacterial suspension after the 3-hour culture. (**b**) The sorting ratio of the mixed bacterial samples after applying 2 MHz and 100 V_pp_ *F_pDEP_*. Each value was calculated from three repetitions. Statistical significance (*) was tested using the Student *t*-test to indicate *p* < 0.001.

As described above, the lysed *E. coli* released cytoplasm to increase the conductivity of the droplet solution due to the bactericidal effect of the Amp after the 3-hour culture. In contrast, AmpR *E. coli* can resist Amp and, thus, do not result in a significant conductivity increase. However, in real-world samples, AmpR *E. coli* most likely coexist within general bacterial samples. Therefore, the mixed bacterial samples with 5, 10, 30, and 50% AmpR *E. coli* were prepared to estimate how these different ratios would affect the droplet-sorting ratios. Figure 6a shows the change in the broth conductivity and the relative growth ratio after culturing the mixed bacteria in 9.4% LCLB/100 μg/mL Amp for 3 h. The conductivity decreased with an increasing AmpR *E. coli* ratio because the AmpR *E. coli* can generate beta-lactamase to break down the Amp structure [38], protecting normal *E. coli* from deterioration. The lower residual Amp permits more *E. coli* proliferation without cell wall breakdown.

The growth ratio curve of Figure 6a exhibits a similar result: the higher AmpR *E. coli* ratio allows the larger bacterial growth ratio even though the broth contains Amp antibiotics. When the AmpR *E. coli* ratio increased to 30%, the growth ratio drastically increased to 117%. This result suggests that the 30% AmpR *E. coli* can effectively digest Amp to protect normal *E. coli* from being susceptible to Amp. Moreover, when the AmpR *E. coli* ratio reached 50%, the growth ratio was almost the same as that of the 100% AmpR. *E. coli* sample, indicating that the 50% AmpR *E. coli* is more than enough to digest the 100 μg/mL Amp to eliminate the Amp inhibition on *E. coli*.

Figure 6b shows the sorting ratio of the ratio-varied AmpR *E. coli*-containing droplets with the 2 MHz *F_pDEP_*. The result indicates that the sorting ratio significantly decreased to about 52% when the AmpR *E. coli* ratio was equal to or higher than 50%. When the AmpR *E. coli* ratio was smaller than 50%, the sorting ratios were 74−81%. This result indicates that the significant change in the sorting ratio can be used as a threshold percentage of AmpR *E. coli* in an unknown sample. If the perpendicular displacement of droplets can be quantified numerically, such as using AI-based image recognition, the AmpR *E. coli* ratio can be distinguished with better sensitivity in the *F_pDEP_*-based chip. Although the conductometry can distinguish the AmpR *E. coli* ratio better than the *F_pDEP_*-based chip, the *F_pDEP_*-based chip is expected to perform high-throughput and sub-nanoliter-volume detection of antibiotic-resistant bacterial cells.

Table 1 shows a comparison between different microfluidics-sorting platforms for bacterial AST. Most of these sorting platforms required fluorescent probes as a label to distinguish AMR bacteria. Moreover, they needed 6–18 h of culture to enhance the optical signals of AMR bacteria. In contrast, our study can directly recognize the threshold percentage of AMR bacteria after the 3-hour culture, which is much shorter than the other sorting platforms.

## 4. Conclusions

This study presents a label-free DEP-based droplet sorter for estimating AMR bacteria by using the droplet-sorting ratio as an index. The sorting component of the droplet-sorter chip can attract the high-conductivity droplets to the H*F_pDEP_* channel. The 9.4% LCLB can adequately maintain *E. coli* growth, allowing Amp to destroy the cell wall synthesis of *E. coli*, which results in a significant increase in conductivity of the low-conductivity medium (by about 100 μS/cm) of the *E. coli*/LCLB/Amp mixture after the 3-hour culture. The *E. coli*/LCLB/Amp and the AmpR *E. coli*/LCLB/Amp-containing droplets exhibit different sorting ratios under the application of 2 MHz and 100 V_pp_ *F_pDEP_*. Moreover, the microfluidic platform can probe the threshold ratio of AmpR bacterial cells in an unknown bacterial sample based on the sorting ratio when the AmpR *E. coli*-to-*E. coli* ratio is equal to or higher than 50%. This conductivity-dependent DEP-based microfluidic sorting platform exhibits promising applications for rapidly detecting the effectiveness of cell wall-targeting antibiotics on AMR bacteria.

## Figures and Tables

**Table 1:** Comparison between different microfluidics-sorting platforms for bacterial AST.

Bacteria in Droplets	Antibiotics	Detection/Label or Label-Free	Culture Time (h)	Reference
*E. coli* HS151	fusidic acid	light scattering/label-free	6–8	[30]
*E. coli* DH5α	cefotaxime	yellow fluorescent protein/label	overnight	[40]
Staphylococcus aureus	cefazolin	SyTox Orange viability dye/label	15–17	[41]
*E. coli* ECJW992	streptothricin F	fluorescent protein/label	24	[42]
*E. coli* MG1655	rifampicin	enhanced green fluorescent protein /label	12–18	[43]
*E. coli* HB101	ampicillin	droplet conductivity/label-free	3	this work

## Data Availability

The data will be made available upon request.

## Supplementary Materials

The following supporting information can be downloaded at https://www.mdpi.com/article/10.3390/bios14050218/s1, Figure S1: The channel design and optical images of droplet generator and sorter; Figure S2: The electrodes and the calibration curve for conductivity measurements; Figure S3: The images of AmpR *E. coli*/LCLB/Amp-containing droplet sorting trajectory.
