# Supplementary material for: A Label-Free Droplet Sorting Platform Integrating Dielectrophoretic Separation for Estimating Bacterial Antimicrobial Resistance
Source: Biosensors (Basel). 2024 Apr 26;14(5):218. doi: 10.3390/bios14050218 (PMC11117925; doi:10.3390/bios14050218)
Supplement: Supplementary file 1 [file biosensors-14-00218-s001.zip › biosensors-2945336-supplementary.pdf]

A Label-Free Droplet Sorting Platform Integrating Dielectrophoretic Separation for Estimating Bacterial Antimicrobial Resistance

Jia-De Yan [1], Chiou-Ying Yang [2], Arum Han [3,4,5,*] and Ching-Chou Wu [1,6,7,*]

[1] Doctoral Program in Tissue Engineering and Regenerative Medicine, National Chung Hsing University, Taichung City 402, Taiwan; e168gg@gmail.com
[2] Institute of Molecular Biology, National Chung Hsing University, Taichung City 402, Taiwan; cyyang@dragon.nchu.edu.tw
[3] Department of Electrical and Computer Engineering, Texas A&M University, College Station, TX 77843, USA
[4] Department of Biomedical Engineering, Texas A&M University, College Station, TX 77843, USA
[5] Department of Chemical Engineering, Texas A&M University, College Station, TX 77843, USA
[6] Department of Bio-industrial Mechatronics Engineering, National Chung Hsing University, Taichung City 402, Taiwan
[7] Innovation and Development Center of Sustainable Agriculture, National Chung Hsing University, Taichung City 402, Taiwan
* Correspondence: arum.han@ece.tamu.edu (A.H.); ccwu@dragon.nchu.edu.tw (C.-C.W.); Tel.: +1-979-845-0686 (A.H.); +886-4-2285-1268 (C.-C.W.)

Supplementary Information

S1. Conductivity measurements

The four electrodes were patterned with Ti (20 nm)/Au (200 nm) ultrathin films on a cleaned glass substrate. A polydimethylsiloxane (PDMS) slab drilled with a container of 3.5 mm diameter was mounted on the four-electrode chip for conductivity measurements, as shown in Fig. S2 (a). The electrode design and the optical image of the detection area are respectively shown in Fig. S2 (b and c). The width of each electrode in the detection region was 100 μm. Two of the electrodes can be chose for different cell constants composition. In this work, the outermost two electrodes were chosen for conductivity measurements with an adequate cell constant in the experiments. Concentration-varied potassium chloride (KCl) solutions were sequentially dripped into the chip container. The LCR meter recorded the resistance from 100 to 100000 Hz with the 0.1 V_{pp}

voltage. The solution resistance taken at 5.0 kHz showed a good linearity with the KCl concentration, as shown in Fig. S2(d). The calibration curve has a good correlation coefficient (R = 0.995).

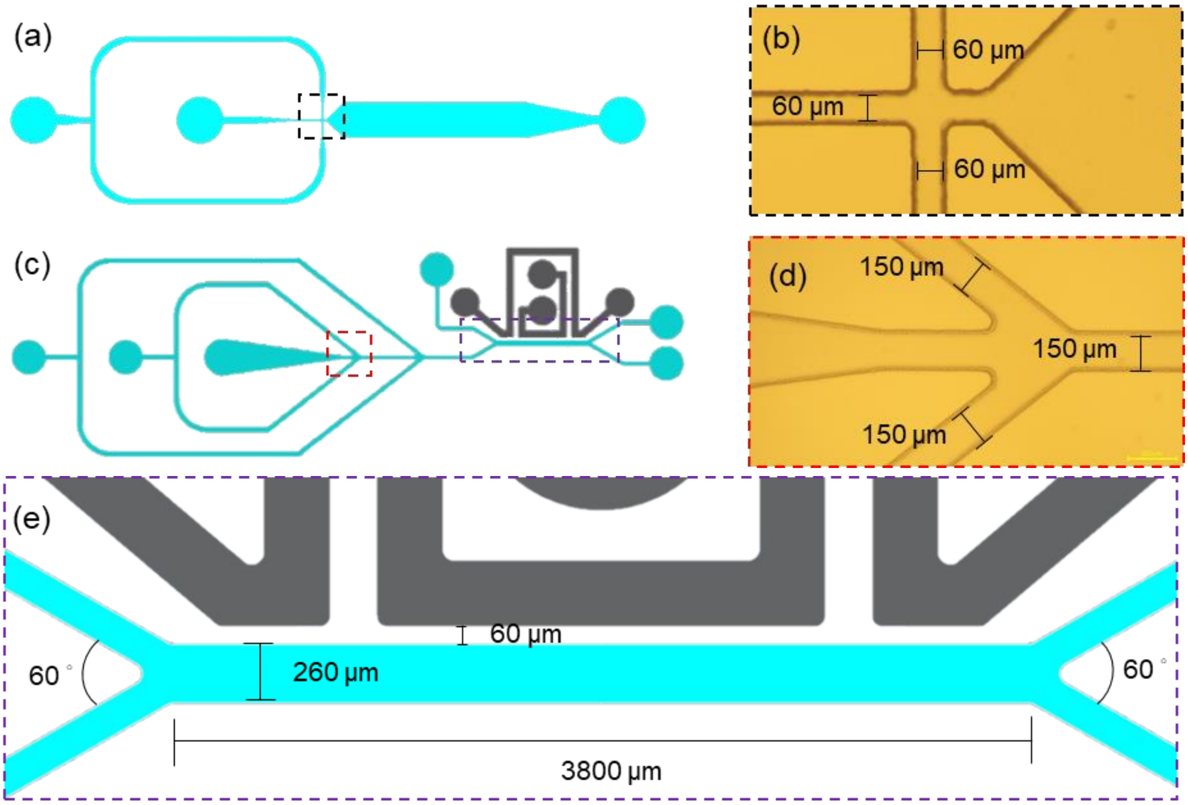

Figure S1 (a) The channel design of the droplet generator. (b) The optical image of the channel intersection in the black region of (a). (c) The design of microchannels and DEP electrodes for spacing and sorting droplets. (d) The optical image of the channel intersection in the red region of (c). (e) The magnification of the DEP-sorting region, consisting of the Y-shaped microchannels (light blue) and the DEP electrode pair (dark gray), in the violet part of (c).

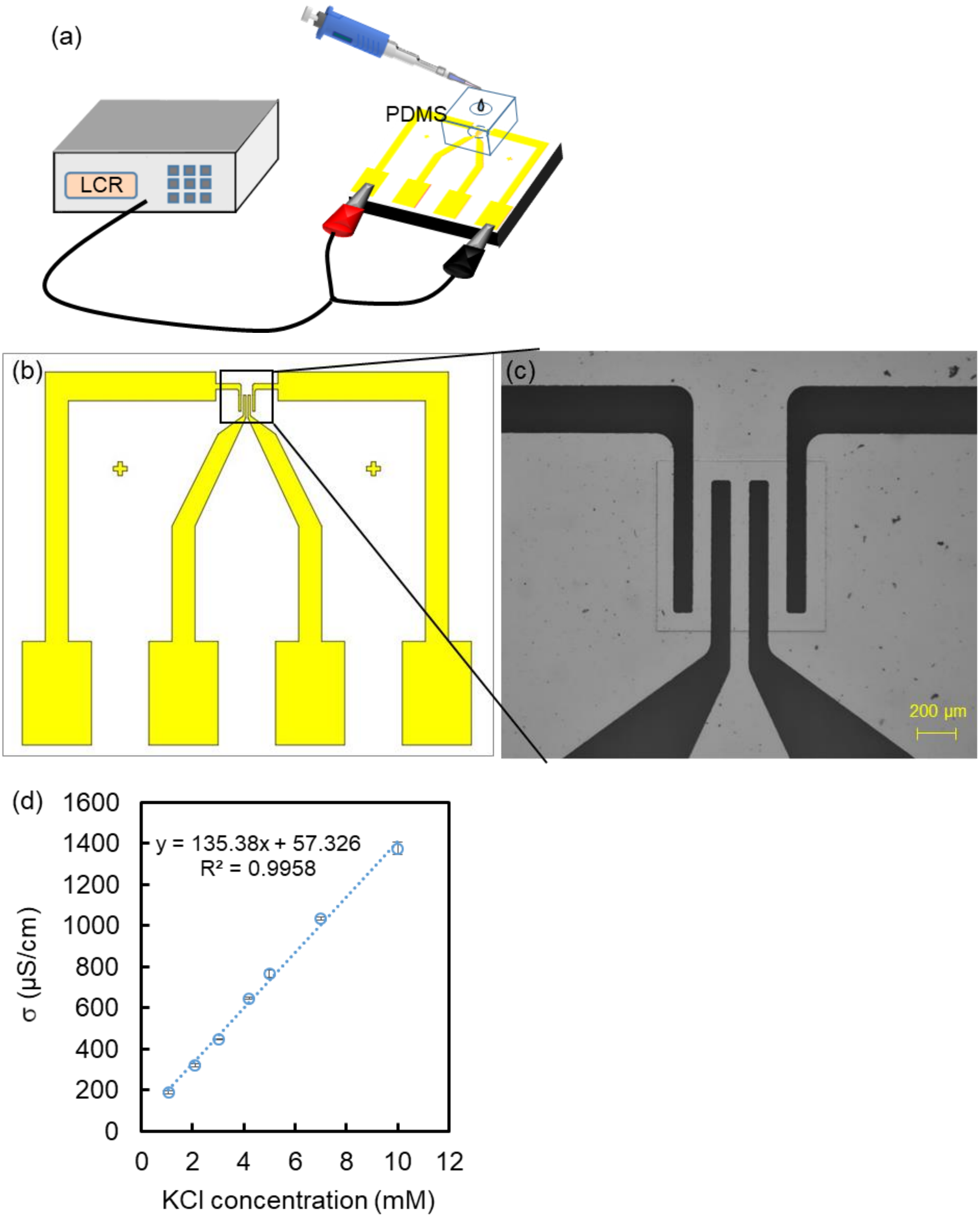

Figure S2 (a) Illustration of the chip operation. (b) Design of the four-electrode chip. (c) Optical image of the chip detection region. (d) The calibration curve of conductivity versus concentration-varied KCl solutions were measured on the chip.

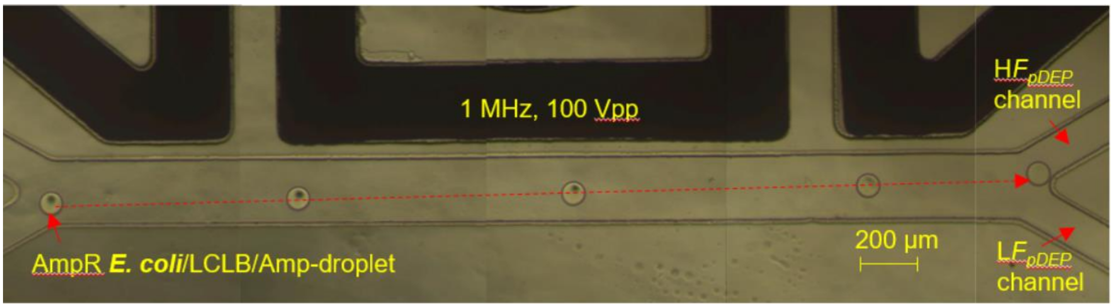

Figure S3 The image stitching to show the trajectory of the AmpR *E.coli*/LCLB/Amp-containing droplet in the channel for sorting. The DEP was applied with 100 V_{pp} at 1 MHz.
